# Modeling adaptive response profiles in a vaccine clinical trial

**DOI:** 10.1186/s12874-020-01070-3

**Published:** 2020-07-16

**Authors:** Dicle Hasdemir, Robert A. van den Berg, Antoine van Kampen, Age K. Smilde

**Affiliations:** 1 Bioinformatics Laboratory, Department of Clinical Epidemiology, Biostatistics and Bioinformatics, Academic Medical Center, University of Amsterdam, Amsterdam, The Netherlands; 2 Biosystems Data Analysis Group, University of Amsterdam, Amsterdam, The Netherlands; 3 GSK, Rixensart, Belgium; 4 Current address: GSK, Rockville, United States

**Keywords:** LMM, Linear mixed model, Vaccine, Adjuvant, AIC, BIC, DIC, Conditional AIC, Random effect selection, Model selection, Quantification of individual differences, Immunology

## Abstract

**Background:**

Vaccine clinical studies typically provide time-resolved data on adaptive response read-outs in response to the administration of that particular vaccine to a cohort of individuals. However, modeling such data is challenged by the properties of these time-resolved profiles such as non-linearity, scarcity of measurement points, scheduling of the vaccine at multiple time points. Linear Mixed Models (LMM) are often used for the analysis of longitudinal data but their use in these time-resolved immunological data is not common yet. Apart from the modeling challenges mentioned earlier, selection of the optimal model by using information-criterion-based measures is far from being straight-forward. The aim of this study is to provide guidelines for the application and selection of LMMs that deal with the challenging characteristics of the typical data sets in the field of vaccine clinical studies.

**Methods:**

We used antibody measurements in response to Hepatitis-B vaccine with five different adjuvant formulations for demonstration purposes. We built piecewise-linear, piecewise-quadratic and cubic models with transformations of the axes with pre-selected or optimized knot locations where time is a numerical variable. We also investigated models where time is categorical and random effects are shared intercepts between different measurement points. We compared all models by using Akaike Information Criterion (AIC), Bayesian Information Criterion (BIC), Deviance Information Criterion (DIC), variations of conditional AIC and by visual inspection of the model fit in the light of prior biological information.

**Results:**

There are various ways of dealing with the challenges of the data which have their own advantages and disadvantages. We explain these in detail here. Traditional information-criteria-based measures work well for the coarse selection of the model structure and complexity, however are not efficient at fine tuning of the complexity level of the random effects.

**Conclusions:**

We show that common statistical measures for optimal model complexity are not sufficient. Rather, explicitly accounting for model purpose and biological interpretation is needed to arrive at relevant models.

**Trial Registration:**

Clinical trial registration number for this study: NCT00805389, date of registration: December 9, 2008 (pro-active registration).

## Background

Antibody levels are indicative of an individuals’ adaptive immune response against a specific antigen. In vaccine clinical trials, these adaptive response readouts are collected in time following vaccine interventions. Such interventions with a certain vaccine induce a time-resolved change in the adaptive response outcome; individuals generally exhibit distinct patterns of immune response in time or reach different levels of protection.

Analyzing and modeling these adaptive response profiles can serve several goals. One of the goals might be to understand the differences in treatment effects, such as the administration of the vaccine using different adjuvants. In that case, the interest is in the differences between groups of subjects which have received different adjuvants. Another goal of such a study might be to establish the magnitude of inter-individual variation of subjects within groups. A third goal of modeling such data can be to quantify individual differences with the purpose of relating those differences to several external variables. In this paper, we will focus on this latter goal.

Often, the inter-individual differences are associated with characteristics of the subjects’ early immune response which manifest themselves in the early days after the vaccination or already in the baseline states of the immune response components. Future vaccine research can highly benefit from discovering early/innate response - adaptive response associations, since these can guide mechanistic studies ultimately leading to more efficacious vaccines and better vaccination schemes. To do this, we have to find good estimates of the adaptive response in the individuals, such that at a later stage these can be associated with early/innate response. A good example of early response is the change in gene expression levels after vaccination. Although modeling such an association can also be done simultaneously, in this paper we focus on finding good estimates of the adaptive response read-outs (hence quantitative measures describing individual responses) as a first step. These estimates can then easily be associated with gene expression data (or yet other covariates) in subsequent analyses as mentioned above.

The need for such estimates is due to variation in clinical data, which can be very high. Data is typically collected once per time point per individual and the measurements are heavily affected by factors such as the time of the day and temperature [1]. Therefore, inter-individual differences arising from such factors can wrongly be attributed to the study factors under investigation. Reducing such unwanted variability can be performed by modeling all individuals collectively and exploiting the shrinkage property of Linear Mixed Models (LMMs). In an LMM, model coefficients consist of a fixed part which is the same for all the individuals (and can, e.g., be used for estimating treatment effects) and a random part which can model the individual differences. Therefore, the model structure allows retaining inter-individual differences in the random coefficients in a robust manner while similarities across individuals are attributed to the fixed part. This approach covers all individuals at once, hence it is not restricted to one individual per model.

LMMs provide a suitable framework for systematic identification and quantification of inter-individual differences. They have already been extensively used in similar experimental setups of growth curve analysis [2] which covers the analysis of longitudinal data. LMMs have also been used previously for the analysis of data originating from vaccine studies [3–5]. In these studies, they were mainly used for prediction of late individual adaptive responses following vaccination (persistence of response). Nevertheless, the use of mixed models in the vaccine field is still far from standard practice. Although principles of growth curve analysis prove useful in the analysis of clinical data for vaccine research [6], specific challenges in modeling adaptive response profiles stem from the infrequent and irregular sampling through a highly dynamic regime with multiple interventions. In this paper, we give guidelines for application of LMMs under such restrictions on the data, where we will explore different types of LMMs that differ in the way they model the factor time. We also discuss model selection and - in that context - the balancing of formal statistical measures and biological interpretation.

**Fig. 1:**
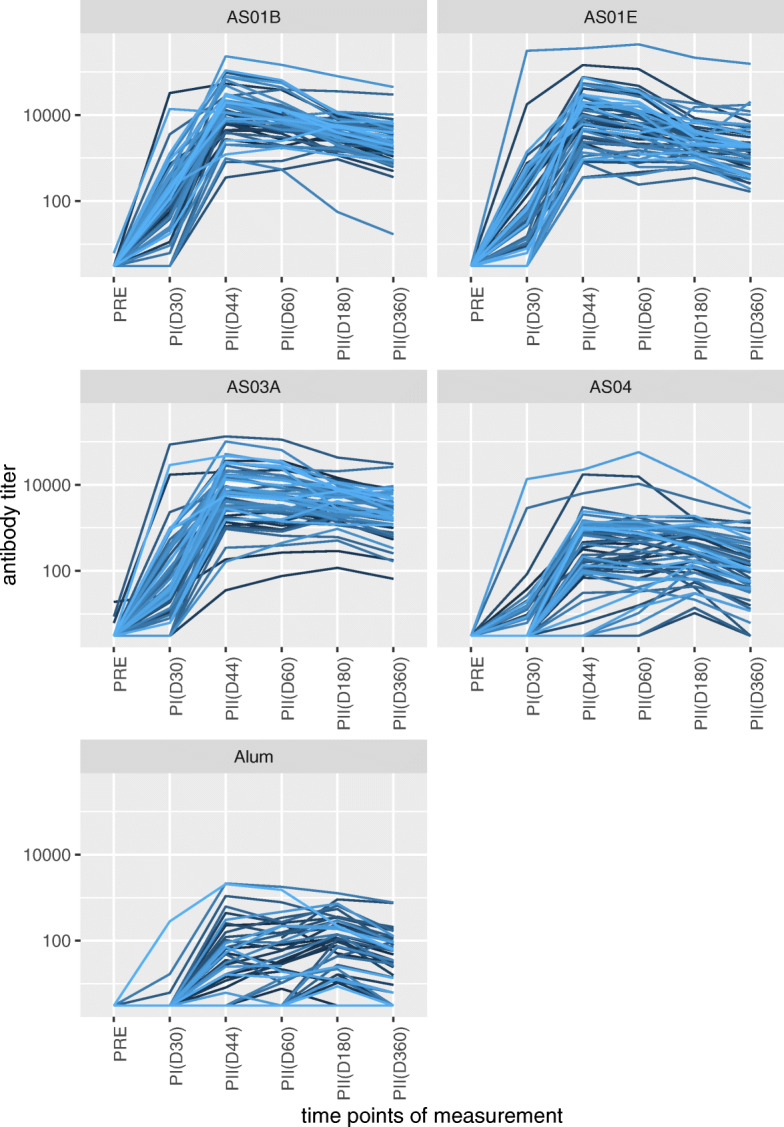
**Time profiles of antibody (Ab) levels.** Each Ab-profile (plotted in a different color from the gradient blue color scale) denotes a subject. The y-axis denotes the antibody levels on log10 scale. The x-axis indicates the measurement points: prior to vaccinations (PRE), Day 30 (PI(D30)), Day 44 (PII(D44)), Day 60 (PII(D60)), Day 180 (PII(D180)) and Day 360 (PII(D360)). PI and PII show the measurements after the first and second vaccinations, respectively. The five panels correspond to different adjuvants (AS01B, AS01E, AS03A, AS04 and Alum) and each group consists of different subjects. The bird’s eye view on Ab levels presented here helps to visualise the increased divergence between the individual responses observed especially in the AS04 and Alum groups

The data used in this study comprises antibody readouts in response to vaccinations with HBsAg surface antigen mixed with five different adjuvants (See [7] for more details of the specific data set). Antibody profiles are shown in Fig. 1 and are the focus of our paper. The five adjuvant groups do not only exhibit distinct group patterns in time but also different degrees of variability across individual responses. The time-resolved responses can be roughly divided in three segments excluding delays: i) a rising response after the first vaccination (between Day 0 (denoted as PRE) and Day 30), ii) a rising response after the second vaccination (between Day 30 and Day 44), iii) a decaying response after Day 44. A biological interpretation would then be in terms of rates of responses during those segments, both at a group and individual level.

Statistical measures that we involve in our paper include Akaike Information Criterion (AIC) [8], Bayesian Information Criterion (BIC) [9], Deviance Information Criterion (DIC) [10], conditional AIC [11] and a variant of conditional AIC [12]. These different measures are necessary since we want to assess model performance both regarding the fixed effects as well as the random effects.

## Methods

### Models

We chose to study the model forms that would be able to address specific characteristics of the data. These included (i) high levels of inter-adjuvant variation with respect to the time point of the maximum response, (ii) the overall time-resolved response pattern, (iii) increased inter-individual variation in some adjuvant groups, (iv) infrequent sampling towards the end of the time trajectory and (v) including the interventions (See Fig. 1).

The model equations used in this study are given in Table 1. The choice of the models presented in Table 1 is the following. Choosing an appropriate functional form for the fixed effect of time is the primary step in modeling longitudinal data in a mixed models framework. In a typical vaccine clinical study in which there are multiple interventions (vaccinations) the immune response does not follow a simple linear behavior with a single slope in time. One way to deal with this is to employ non-linear regression. In that approach, it is assumed that an exact mathematical formula defines the underlying immune response process. This provides us with biologically interpretable parameters but that approach is prone to problems associated with model convergence [13] and requires solid assumptions about the underlying model and therefore is not practical from a data analysis point of view. Furthermore, it is not clear how to model the interventions.

**Table 1:** **Model equations**

2-segment piecewise (PW) linear	*Y*_*ij*_=*β*_0*i*_+*β*_1*i*_.*t*_*j*_+*β*_2*i*_.(*t*_*j*_−*t*_*KNOT*_)_+_+*ε*_*ij*_
	*β*_0*i*_=*γ*_00_+*ζ*_0*i*_
	*β*_1*i*_=*γ*_10_+*ζ*_1*i*_
	*β*_2*i*_=*γ*_20_+*ζ*_2*i*_
	i: subject index, j: time index
	(*t*_*j*_−*t*_*KNOT*_)_+_ is a derived variable which becomes (*t*_*j*_−*t*_*KNOT*_) only when *t*_*j*_>*t*_*KNOT*_. Otherwise it is 0.
	*γ*_00_ and *ζ*_0*i*_ are fixed and random intercepts
	*γ*_10_ and *ζ*_1*i*_ are fixed and random first segment slopes
	*γ*_20_ and *ζ*_2*i*_ are fixed and random incremental slopes
3-segment PW linear	*Y*_*ij*_=*β*_0*i*_+*β*_1*i*_.*t*_*j*_+*β*_2*i*_.(*t*_*j*_−*t*_*KNOT*1_)_+_+*β*_3*i*_.(*t*_*j*_−*t*_*KNOT*2_)_+_+*ε*_*ij*_
	*β*_3*i*_=*γ*_30_+*ζ*_3*i*_
	*γ*_30_ and *ζ*_3*i*_ are also fixed and random incremental slopes
Cubic	$Y_{ij} = \beta _{0i} + \beta _{1i}. t_{j} + \beta _{2i}. t_{j}^{2} + \beta _{3i}. t_{j}^{3} + \epsilon _{ij}$
	*γ*_00_ and *ζ*_0*i*_ are fixed and random intercepts
	*γ*_10_ and *ζ*_1*i*_ are fixed and random first order coefficients
	*γ*_20_ and *ζ*_2*i*_ are fixed and random second order coefficients
	*γ*_30_ and *ζ*_3*i*_ are fixed and random third order coefficients
2-segment PW quadratic	$Y_{ij} = \beta _{0i} + \beta _{1i}. (t_{j} - t_{KNOT})_{+} + \beta _{2i}. t_{j}^{2} + \beta _{3i}. (t_{j} - t_{KNOT})_{+}^{2} + \epsilon _{ij}$
	*γ*_00_ and *ζ*_0*i*_ are fixed and random intercepts
	*γ*_10_ and *ζ*_1*i*_ are fixed and random first order coefficients
	*γ*_20_ and *ζ*_2*i*_ are fixed and random second order coefficients
	*γ*_30_ and *ζ*_3*i*_ are fixed and random incremental second order coefficients
time as a categorical variable	*Y*_*ij*_=*γ*_00_.*D*_0_+(*γ*_10_+*ζ*_1*i*_).*D*_1_+(*γ*_20_+*ζ*_2*i*_).*D*_2_+(*γ*_30_+*ζ*_2*i*_).*D*_3_+(*γ*_40_+*ζ*_3*i*_).*D*_4_+(*γ*_50_+*ζ*_3*i*_).*D*_5_+*ε*_*ij*_
	*γ*_00,10,20,30,40,50_ are fixed coefficients
	*ζ*_1*i*,2*i*,3*i*_ are random coefficients shared between time points
	*D*_0,1,2,3,4,5_ are dummy indicator variables for time points

Polynomial time effect models are linear in the parameters in contrast to the non-linear regression approach. If we assume that the change in time can be approximated with small increases and decreases of constant slopes in small time intervals, a piecewise linear regression approach would suffice. Also cubic models is a viable alternative. Another alternative encountered in vaccine research is using time as a categorical variable. Models developed along these lines are shown in Table 1 and may serve the purpose of this study. They are approximately ordered in increasing complexity and it is expected that the more complex model increases model fit. This comes, however, at the cost of unstable parameter estimates and decreased biological interpretation. Hence, we had to strike a balance and that is the main theme of this paper. We used several information-criterion-based models to judge the statistical performance of the models.

### Estimation and validation

We fitted the models by maximizing the likelihood (ML) of the parameters given the data unless otherwise stated. In some cases we used maximizing the residual likelihood (REML) because that method gave better results for estimating the random effects for some of the models; we will indicate this in the text. We have used plots to illustrate fit of the fixed and random effects. Biological interpretation was judged according to the principles outlined in the introduction.

The well known AIC is based on the marginal likelihood value and was originally developed considering fixed effect models. However, when the focus is on the inference of random effects, calculating the effective degrees of freedom is not straightforward. Therefore, different measures have been proposed for random effects selection. The Bayesian-rooted Deviance Information Criterion (DIC) [10, 14] or information criteria based on conditional likelihood [11] are the most referenced in the literature. This is the reason why we included these measures next to the marginal AIC in our study. We have also included Bayesian Information Criterion due to its well known advantages in punishing complexity.

We chose to make a model per adjuvant group and not across all groups simultaneously. The latter would amount to using dummy variables to encode the different groups and subsequently fit an overall model. Due to the considerable differences of effects between the groups, interaction terms describing these differences would be needed in such an approach. This would complicate the analyses considerably and we therefore decided to model each group separately, since the sample sizes per group were large enough. Consequently, the reported AIC values cannot be compared across groups, only within groups, because within groups the different models are fitted on the same data whereas this is not the case between groups.

We carried out all calculations using R [15] with extensive use of the lme4 package [16] for fitting the models. We used also cAIC4 package for the calculation of the corrected conditional AIC. All scripts can be provided through communication with the authors.

## Results

### Piecewise linear models with fixed knot location across all groups

The antibody levels data in this study are roughly characterized by an increase due to vaccination and a subsequent decrease in time (Fig. 1). Therefore, the simplest functional form we considered was a 2-segment piecewise form with a fixed knot location. The two segments were connected at the day of maximum response (Day 44 based on prior knowledge). Consequently, the first segment covers PRE to Day 44, while the second segment covers Day 44 to Day 360.

Visual inspection of the 2-segment model showed that it performed relatively well in the first three adjuvant groups (AS01B, AS01E, AS03A). This is rather expected because most of the individuals treated with these adjuvanted vaccines don’t experience a delay in response after the first vaccination (Fig. 1). However, even in these groups the measurements at Day 30 were consistently overestimated because this model structure cannot reflect the difference between the rates of antibody level increase after the first and second vaccination. This resulted in AIC’s ranging from 827 to 1254 (see Table 2 row 1).

**Table 2:** **Marginal AIC values of models where time axis is numerical.** In the case of models with optimized knot location(s), we show the AIC’s of models with the lowest AIC amongst models of the same complexity with different knot locations. All AIC’s calculated in this case can be seen in the Supplementary Tables and are calculated using ML estimation. The cells with an NA show unbuilt models. This is because knot optimization in 2-segment first order polynomial models of these adjuvants did not result in much gain, unlike the last two adjuvants (see first and third rows), therefore we skipped knot optimization in the 3-segment models for those. Models in the last two rows were again built for all adjuvants due to their expected good performance

Fixed Effect Structure	Random Effect Structure	Model specification	Adjuvant				
			AS01B	AS01E	AS03A	AS04	Alum
2-segment 1^*st*^ order polynomial	2 slopes	knot at Day 44	1179.27	1228.55	1189.45	1254.54	827.64
3-segment 1^*st*^ order polynomial	3 slopes	knots at Days 30 & 44	767.99	796.35	723.94	865.89	675.69
2-segment 1^*st*^ order polynomial	2 slopes	knot optimized	1175.51	1223.95	1179.99	1216.55	784.10
3-segment 1^*st*^ order polynomial	3 slopes	knots optimized	NA	NA	NA	865.89	669.64
3^*rd*^ order polynomial	all degrees except intercept	x-axis transformed	1385.02	1389.77	1344.64	1327.85	822.13
2-segment 2^*nd*^ order polynomial	all degrees except intercept	x-axis transformed, knot optimized	1189.33	1227.23	1181.72	1210.15	765.78

### Piecewise linear models with optimized knot locations

With a grid-search we found that the best 2-segment PW model fits for the AS01B, AS01E and AS03A groups located the knot (and hence the time of maximum response) between Days 45-48. For AS04, this location was around Day 57 and for the Alum group, it was around Day 75 (see Table 2 row 3 and Table S1). The AIC’s improved well for the AS04 and Alum groups unlike the other groups compared to the respective AIC’s with fixed knot models (Table 2 rows 1,3). That can also be seen from the fact that the knot optimization does not change the original fixed knot location Day 44 much for the AS01B, AS01E and AS03A groups.

We focused further on the AS04 and Alum groups because they benefited the most from knot optimization for the 2-segment models we discussed in the previous paragraph. We used a 3-segment linear PW model for that group with again a grid search but now for two knot locations (see Tables S2 and S4). Our grid search revealed that the optimal knot locations were around Days 39 & 42 and Days 33 & 45 for AS04 and Alum respectively. The models were fitted using REML during the grid search because that estimation method gave better performance than ML in finding the optimal knot locations. After having found these optimal knot locations, the models were refitted using ML (given those optimized knot locations) to make the marginal AIC results (see Table 2) comparable.

The AIC of the optimal Alum model was 669 which slightly improved on the fixed knot location 3-segment linear PW model (AIC=675). The AIC of the AS04 model did not improve at all on the respective fixed knot model (Table 2 rows 2,4). The values we obtained show that models did not improve with knot optimization in the 3-segment models unlike the previous 2-segment models. This is apparent also from the visualization of the fits (Fig. 3).

### Cubic and piecewise quadratic models

A third order polynomial appears to be a priori a reasonable functional form of the time effect. By including the third order term also in the random effects, we should be able to capture also some of the inter-individual dynamic response differences. However, the best fits of this form exhibited a large decrease and subsequent increase between D180 and D360 (data not shown). One would not expect this from the underlying biology. In this time interval, we only expect a decay or stabilization in the response. These fits occurred due to infrequent sampling around this time interval. A square root transformation of the time axis may solve this issue by accounting for a more even sampling frequency.

Third order polynomial regression with the time axis square-root transformation can to some extent model dynamic differences and maximum response points between adjuvant groups and model as well prolonged delays in the initial phase (Fig. 4). However, it failed to model steep increases well as reflected both visually and also in the AIC values which became worse or improved only slightly (see Table 2 row 5) compared to the basic 2-segment linear fixed knot models. An important observation from Fig. 4 is the steep decrease of the model that takes place after the end of the first year.

**Fig. 2:**
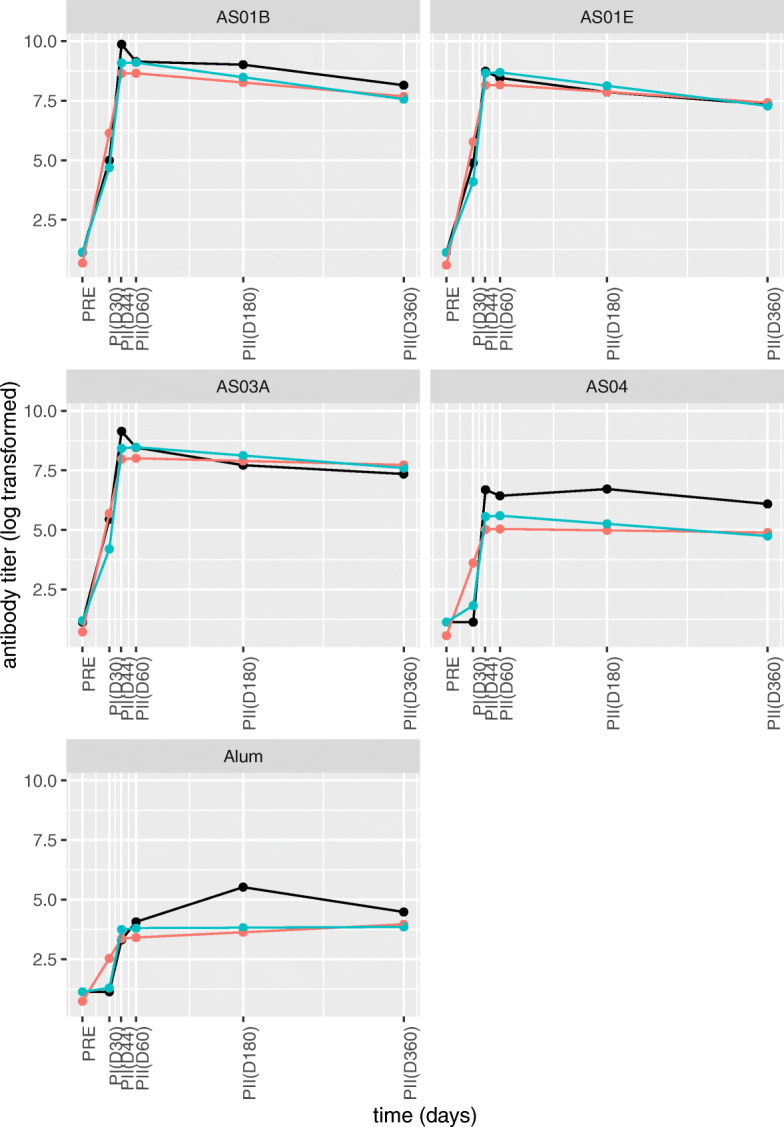
**Population estimates for the 2-segment and 3-segment PW linear models with a-priori fixed knot locations.** Black lines show the time profile (interconnected data points) of an individual. Red and blue lines (estimates interconnected at the measurement points) show the population estimates obtained by the 2-segment and 3-segment PW linear models with a-priori fixed knot locations. These 5 individuals are typical examples of their adjuvant group. Therefore, the population estimates obtained by the models (time profile prediction obtained by using only the fixed part of the model) should reflect the example individual time profile

**Fig. 3:**
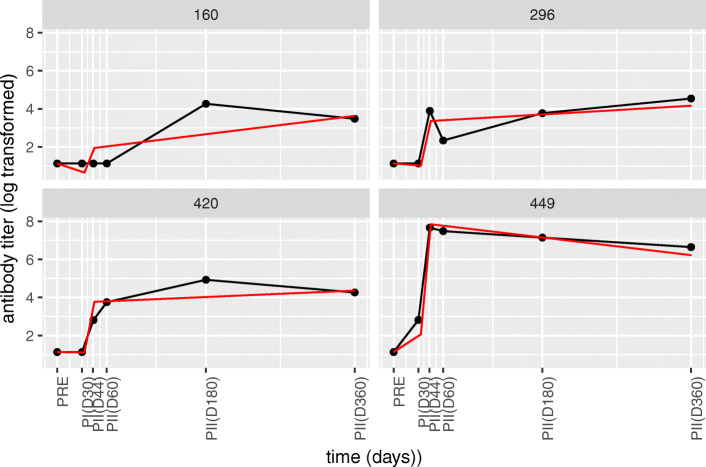
**Selected fits from the Alum group with optimized knot locations for the 3-segment first order models.** Each panel represents an individual from the Alum adjuvant group that was selected for demonstrative purposes. Red lines indicate the model fit and the black lines connect the measured data points. Individuals 160 and 420 are examples whose final line segment, starting from D45, still show an increasing response due to insufficiency of the model structure used. Individual 449 is an example of a good model fit. Overall, the graph shows inter-individual variation in the Alum group

**Fig. 4:**
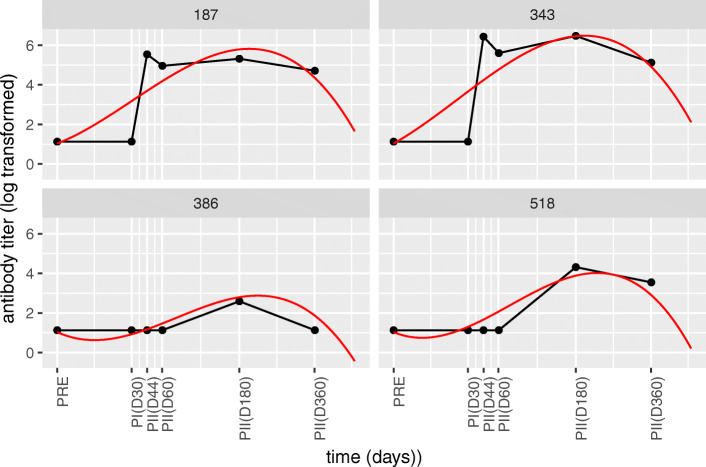
**Selected cubic model fits of individuals from the Alum group.** Each panel represents an individual from the Alum adjuvant group that was selected for demonstrative purposes. Red lines indicate the model fit and the black lines connect the measured data points. The model is inadequate to fit the steep increase in response for individuals 187 and 343. The model is able to correctly fit the prolonged delay in response shown for individuals 386 and 518

**Fig. 5:**
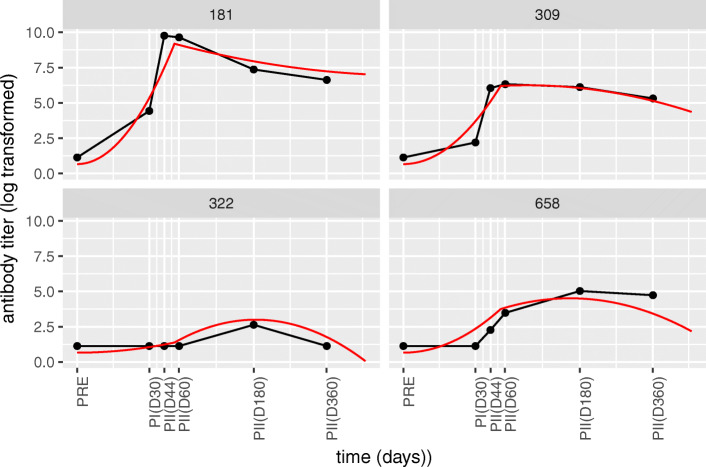
**Selected fits of individuals fits from the AS04 group with quadratic piecewise models.** Each panel represents an individual from the AS04 adjuvant group that was selected for demonstrative purposes. Red lines indicate the model fit and the black lines connect the measured data points. Individuals 181, 309 and 658 are examples where both steep increases and slight decreases with different maxima across individuals are handled well by the model. Individual 322 shows a case in which a prolonged delay was handled well

AIC-wise all adjuvant quadratic piecewise models improved on the cubic models when a 2-segment quadratic form was used. For the AS04 and Alum groups, the AICs were lower also than the 2-segment PW models (Table 2). On top of that the piecewise quadratic models successfully captured the subtle dynamic differences between individuals (see Fig. 5). However, like in the cubic case, this was achieved only after the transformation of the time axis, which makes the interpretation of the parameters difficult. In addition to that, AICs were poorer than the 3-segment PW models which shows that the complexity brought by adding quadratic terms were not needed from the statistical point of view. Visual inspection of the models, however, support the biological plausibility of the higher order terms indicating the discrepancy between statistical and biological model selection criteria.

### Time as categorical variable

Another solution to account for even more subtle dynamic differences is to include time not as a continuous but as a categorical variable. This is the ultimate level of flexibility of modeling (fixed) time effects. Its advantage compared to a quadratic piecewise function is that the time axis does not have to be transformed. With time as categorical variable in the fixed effects, the random effect strusture can be modeled in different ways with increasing complexity. The simplest random effect structure would give one random intercept per person across all time points simultaneously, whereas the most complex structure would allow a random intercept per time point per person. The most complex random effect structure would absorb all degrees of freedom (perfect fits) and does not allow for the shrinkage effect to take place. Hence, we restricted some of the time points to share the same intercept instead of a separate intercept per time point. Determining which time points to lump together in this way pointed again at the necessity of a detailed analysis of the trade-off between model complexity and biological interpretability. We used conditional AIC, DIC and BIC to judge model performance in addition to the marginal AIC, as we were interested (also) in the random effects and those have been proposed in the literature for this specific purpose [10, 11].

The optimal model selected based on all measures was almost always the most complex model (Tables S6, S7, S8, S9 and S10). One exception to that is for the Conditional Corrected AIC measure for AS01E model selection (Table S10). However, we do not consider it an advantage to use this measure over the others since in general this measure also followed the same trend with others. Another exception is that the model that was chosen for AS03A adjuvant group by BIC aggreed with our final model selection (Table S8). In this case, BIC was successful in penalizing the complexity sufficiently. However, that did not hold in all cases and therefore, we can’t advise relying solely on BIC for model selection either.

We did not test models with a random intercept at *t*_0_ due to very small inter-individual variation at that specific time point. Table 3 shows the Conditional AIC [11] values of the models with the smallest AIC and the competing models with the closest AIC to it (for a more detailed account, see Table S7). Very small AICs and the perfect model fits with the most complex model in the AS04 and Alum adjuvant groups (even negative AIC’s!) were due to the fact that all individuals were at the same level at *t*_0_ in these groups. Therefore, a model with different random intercepts at each time point except *t*_0_ is practically of such a high flexibility level that the model is not meaningful anymore (for a plot of the fit of these models, see Fig. S1).

**Table 3:** **Conditional AIC values of the categorical models [**11**].** The abbreviations used for the model form denote the random intercepts included in the model. For example, *t*_1_ denotes a random intercept specific to the first time point whereas *t*_45_ denotes a random intercept which is common in the fourth and fifth time points

		AS01B	AS01E	AS03A	AS04	Alum
model with the smallest AIC	model form	*t*_1_,*t*_2_,*t*_3_, *t*_4_,*t*_5_	*t*_1_,*t*_2_,*t*_3_, *t*_4_,*t*_5_	*t*_1_,*t*_2_,*t*_3_, *t*_4_,*t*_5_	*t*_1_,*t*_2_,*t*_3_, *t*_4_,*t*_5_	*t*_1_,*t*_2_,*t*_3_, *t*_4_,*t*_5_
	AIC	-430.71	48.14	139.24	-8176.82	-6394.57
model with the second smallest AIC	model form	*t*_1_,*t*_2_, *t*_3_,*t*_45_	*t*_1_,*t*_23_, *t*_4_,*t*_5_	*t*_1_,*t*_2_, *t*_3_,*t*_45_	*t*_1_,*t*_23_, *t*_4_,*t*_5_	*t*_1_,*t*_2_, *t*_3_,*t*_45_
	AIC	-81.92	55.35	196.20	213.96	193.40

For moderating the trade-off between complexity and biological interpretability, we then used an alternative approach based on tracking the model fit and complexity in the light of biological prior information. Our approach includes a measure of model fit like all information criteria, namely normalized sum of squared residuals, and a penalization method for complexity. In our penalization framework, we allow an increase in the complexity of the model only if the increase in fit is relatively high compared to the previous less complex model in the series. During this process, we also are very cautious in obtaining biologically interpretable model coefficients in terms of the three qualitatively different segments as discussed earlier.

**Fig. 6:**
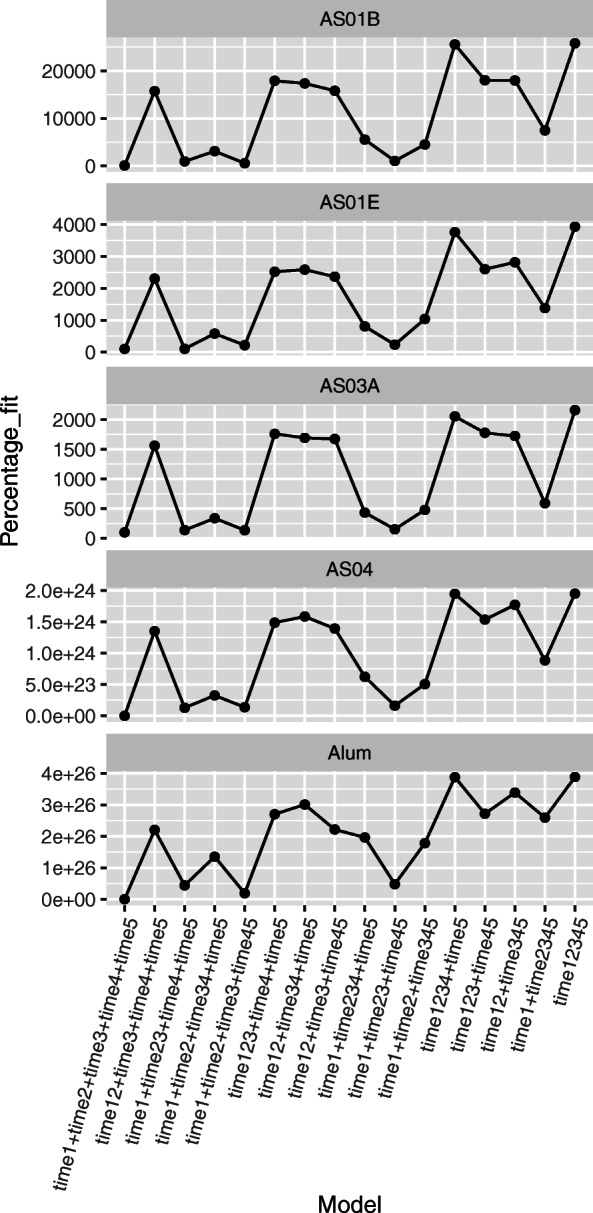
**Model fit for all groups.** The y-axis shows the normalized sum of squared residuals from the fitted data for all groups separately. The value is always normalized to the sum of squared residuals from the first model of each panel. The x-axis shows the models with random point-wise intercepts. For example, model t12+t3+t4+t5 shows a model with no random intercept at *t*_0_, a shared random intercept at *t*_1_ and *t*_2_, and different intercepts at *t*_3_, *t*_4_ and *t*_5_. The first model is always the most flexible model with separate random intercepts for *t*_1_, *t*_2_, *t*_3_, *t*_4_ and *t*_5_

Following this protocol, we first plotted the degree of fit as in Fig. 6. The figure shows how adequate the model fit is at random intercept models with decreasing complexity (from left to right in each panel). A striking observation from this figure is as follows: Models with shared intercepts at either *t*_2_ and *t*_3_ (e.g. the third from the left in each panel) or *t*_4_ and *t*_5_ (e.g. the fifth from the left in each panel) resulted in very good fits (see also row 2 of Table 3). Furthermore, when both shared intercepts were used (e.g. the tenth from the left), the model still gave comparably good fit despite of a loss in flexibility. We therefore concluded that the model which imposes both shared intercepts was a plausible model.

## Discussion

### Piecewise linear models with fixed knot location across all groups

The functional form with two segments allowed us to use the time variable in its original units (days) and therefore, biological interpretation of the two slopes were straightforward: the first segment describes the overall response after vaccination and the second segment the long term response. Moreover, if further fixed predictors other than time are simultaneously added to the model (such as cytokine levels measured as part of the early response), interpretation of these additional effects would be easier with such simple model structures (such as the effect of cytokines on the intercept or on the first slope).

To account for the over-estimation problem with two segment models, a 3-segment piecewise (PW) linear form was used with the first knot being at Day 30 (administration of the second vaccination) and the second one at Day 44. The 3-segment PW linear form was sufficient in making the distinction between the different rates of increase after the two vaccinations. Especially for the AS04 and Alum adjuvant groups, this is an improvement since it can model the delay in response which starts at Day 30. The improvement in the fit between the 2-segment and 3-segment models can be seen in Fig. 2 and is supported by a significant drop in the AIC’s (see Table 2 row 2).

### Piecewise linear models with optimized knot locations

Not all adjuvant groups shared the same key characteristics in terms of their adaptive response. Especially the maximum antibody response for AS04 and Alum was often observed at later time points in comparison with the other adjuvants. For several individuals the maximum response even occurred at Day 180. The first step in reflecting such differences in response dynamics between individuals vaccinated with different adjuvants is to build a separate model (with different knot locations) per adjuvant group.

Another biological expectation that we wanted our models to capture was that the time point of maximum response was most likely not to coincide with a certain measurement point. This time point may be in-between two measurement points and this will also differ across the adjuvant groups. To be more specific, it varies even across individuals within the same group. To account for the differences between the adjuvant groups, we fitted a series of models with different knot locations and identified the optimal knot locations with a time-grid for the fixed part of the model.

While the optimized knot locations clearly show that for the last two groups the time of maximum response was delayed, one should still be cautious with its interpretation because the model structure cannot describe the differences between maxima of the individual profiles within the groups. Especially in the Alum group, the individual differences in this respect are very large.

We have to be careful that a change in the knot locations brings a change in the interpretation of the parameters. For example in the Alum group, very low estimated slopes of the first line segment show the delay in the response. The slopes of the second line segment correspond to the rate of increase in the response whereas the final segment is not necessarily a decrease in the response contrary to the AS01B, AS01E and AS03A adjuvant groups (See example individuals in Fig. 3). Another important observation made regarding both Alum and AS04 groups is that many candidate knot locations resulted in comparable model fits due to the increased inter-individual variation (Tables S2, S3 and S4).

We conclude that inter-group differences can be modeled to some extent by using optimized knot locations, leading to a differentiated interpretation of the model parameters. Irrespective of the exact knot locations, 3-segment models have the smallest AIC’s in all adjuvant groups and, therefore, have to be preferred over the other models statistically. However, biological expectations on the general dynamic response profiles (which should be more smooth) and the need of describing inter-individual differences better indicated that other model forms might be suitable.

### Cubic and piecewise quadratic models

Contrary to the higher AIC’s (see Table 2 row 5), the cubic functional form is more biologically relevant than a piecewise linear approach. The reason is that in a piecewise linear approach, we assume a fixed slope in certain time intervals. However, when higher order polynomials are used, we allow smoother decrease after the maximum response point due to lack of intervention in that specific time frame which seems a more reasonable behavior. However, a major drawback associated with the approach is that the time axis transformation makes biological interpretation more difficult because rates of change are now expressed in terms of square-root of time.

It is also possible to capture more subtle important dynamic properties which the current model failed to do. Such an important dynamic feature is the characteristics of the antibody level increase. The slow increase (sometimes even with a delay in response) after the first vaccination and the relatively faster increase after the second vaccination can be better modeled with a concave upward increase, which tends to become more apparent later in time. Modeling such a concave upward increase together with an appropriate delay in time brought the necessity of using piecewise higher order polynomials. We used second order polynomial piecewise regression for this purpose. A piecewise quadratic functional form requires the selection of the optimal knot just like the piecewise linear regression. However, this time the knot is not restricted to be the time of maximum response as a result of the quadratic behavior of the model, which allows a maximum to occur before of after the knot (Fig. 5).

### Time as categorical variable

As shown in the results section, the models selected by formal selection criteria were the most complex models that could be obtained and a biological interpretation along the lines of the three segments (first rise, second rise and long term effect) were very difficult. Moreover, they were far from serving as reasonable models with an observable shrinkage effect. In other words, none of them were successful in penalizing complexity. These observations showed that there might be cases that render the proposed information-criterion-based measures for optimal random effect selection insufficient.

**Fig. 7:**
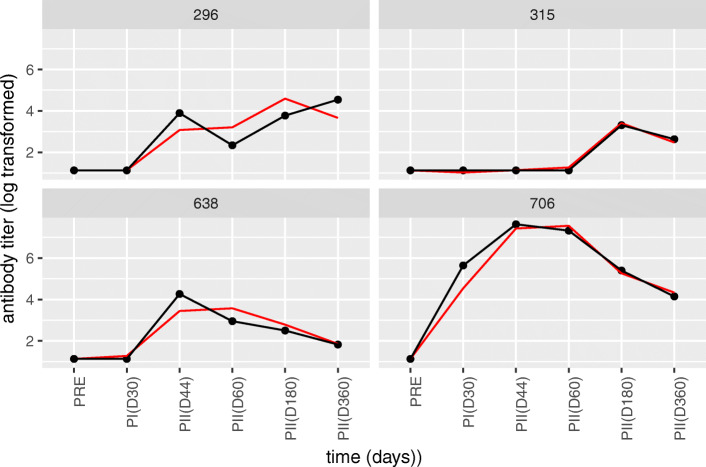
**Selected categorical model fits for four individuals from the Alum group.** Red lines indicate the model fit and the black lines connect the measured data points. Individual 315 shows a too perfect fit. Similarly, the other individuals still show very good fits, much better than the numerical models

Our biologically-guided model selection approach raised less complex models as adequate. For the model selected by this approach, the biological meaning of the random parameters make perfect sense and corresponds nicely with the earlier mentioned three phases in the response: after the first vaccination, in the early phase after the second vaccination and in the late phase after the second vaccination, for the random intercepts at *t*_1_, *t*_2,3_ and *t*_4,5_, respectively. Visual inspection of the plots of this model (Fig. 7) show that the selected model is reasonable (see also the AIC values in Table S6 for a comparison with previous models which considered time as continuous). We should note that this level is still very flexible and therefore, the shrinkage effect is less apparent compared to the previously discussed model structures which consider time as a continuous variable.

## Conclusions

In this paper, we presented a detailed analysis of how different models can be used in linear mixed modeling of adaptive response profiles in clinical vaccine trials which are typically characterized by infrequent and irregular sampling through a highly dynamic regime with multiple interventions. To account for infrequent sampling, we started with very simple models and increased complexity step by step. To account for irregularity in the time axis, we studied model forms with time axis transformation and model forms which consider time as a categorical variable.

Our emphasis was in obtaining good estimates of the individual differences by the LMMs to use those estimates in subsequent models of innate/early response and adaptive response associations. This would provide us with a good way of modeling all subjects collectively and therefore obtaining correct interpretations of the inter-individual variation in the data. We sought model forms that were complex enough to tackle issues in the data. At the same time, we opted for models that were simple enough to result in biologically interpretable parameters. We have concluded that traditional marginal AIC is a good selection measure of fixed effect terms at a coarse level. However, success of measures previously proposed for the fine tuning of random effects, such as variants of conditional AIC and DIC in selecting biologically interpretable models were problematic and, therefore, adding biological information in the selection process is critical. Ultimately, we based our model selection on penalizing the model fit visually in the light of prior biological information.

For the current data, we tend to prefer the model with time as categorical variable and with the restrictions on the random effects as shown in Fig. 7. This seems to be the best compromise between fit and interpretability for this data.

## Supplementary information

**Additional file 1** Supplementary Material.

## Data Availability

The results summary for this study (GSK study number 112115 - NCT00805389) is available on the GSK Clinical Study Register and can be accessed at www.gsk-clinicalstudyregister.com. For interventional studies that evaluate our medicines, anonymized patient-level data will be made available to independent researchers, subject to review by an independent panel, at www.clinicalstudydatarequest.com within six months of publication. To protect the privacy of patients and individuals involved in our studies, GSK does not publically disclose patient- level data.

## Abbreviations

LMM: Linear mixed model
AIC: Akaike information criterion
BIC: Bayesian information criterion
DIC; Deviance information criterion; PRE: Day 0 before vaccination
ML: Maximum likelihood
REML: Residual maximum likelihood

## References

[1] Li S, Rouphael N, Duraisingham S, Romero-Steiner S, Presnell S, Davis C, Schmidt DS, Johnson SE, Milton A, Rajam G (2014). **Molecular signatures of antibody responses derived from a systems biology study of five human vaccines**. Nat Immunol.

[2] Mirman D, Dixon JA, Magnuson JS (2008). **Statistical and computational models of the visual world paradigm: Growth curves and individual differences**. J Mem Lang.

[3] Verbeke G, Molenberghs G: *Linear mixed models for longitudinal data*: Springer; 2009.

[4] Coursaget P, Leboulleux D, Soumare M, le Cann P, Yvonnet B, Chiron J-P, Coll-Seck A-M, Diop-Mar I (1994). **Twelve-year follow-up study of hepatitis B immunization of Senegalese infants**. J Hepatol.

[5] Gilks W, Wang C, Yvonnet B, Coursaget P: **Random-effects models for longitudinal data using Gibbs sampling**. *Biometrics*1993:441–53. 8369380

[6] Mirman D: *Growth curve analysis and visualization using R*: CRC Press; 2016.

[7] Leroux-Roels G, Marchant A, Levy J, Van Damme P, Schwarz TF, Horsmans Y, Jilg W, Kremsner PG, Haelterman E, Clément F (2016). **Impact of adjuvants on CD4+ T cell and B cell responses to a protein antigen vaccine: results from a phase II, randomized, multicenter trial**. Clin Immunol.

[8] Akaike H: **Information theory and an extension of the maximum likelihood principle**. In *Selected Papers of Hirotugu Akaike*: Springer; 1998:199–213.

[9] Schwarz G (1978). **Estimating the dimension of a model**. Ann Stat.

[10] Spiegelhalter DJ, Best NG, Carlin BP, Van Der Linde A (2002). **Bayesian measures of model complexity and fit**. J R Stat Soc Ser B Stat Methodol.

[11] Vaida F, Blanchard S (2005). **Conditional Akaike information for mixed-effects models**. Biometrika.

[12] Greven S, Kneib T: **Marginal and conditional Akaike information criteria in linear mixed models**.

[13] Scheepers C, Keller F, Lapata M (2008). **Evidence for serial coercion: A time course analysis using the visual-world paradigm**. Cogn Psychol.

[14] Bolker BM, Brooks ME, Clark CJ, Geange SW, Poulsen JR, Stevens MHH, White J-SS (2009). **Generalized linear mixed models: a practical guide for ecology and evolution**. Trends Ecol Evol.

[15] R Core Team (2014). R: A Language and Environment for Statistical Computing.

[16] Bates D, Mächler M, Bolker B, Walker S (2015). **Fitting linear mixed-effects models using lme4**. J Stat Softw.

