# Supplementary material for: Modeling adaptive response profiles in a vaccine clinical trial
Source: BMC Med Res Methodol. 2020 Jul 16;20:191. doi: 10.1186/s12874-020-01070-3 (PMC7364493; doi:10.1186/s12874-020-01070-3)
Supplement: Supplementary file 1 — Additional file 1 Supplementary Material. [file 12874_2020_1070_MOESM1_ESM.pdf]

Supplementary Material

Table S1: **Marginal AIC values for 2-segment first order polynomial models.** The models we have chosen are in bold fonts.

Knot (in months)	Knot (in days)	Adjuvant				
		AS01B	AS01E	AS03A	AS04	Alum
1	30	1470.05	1466.57	1480.69	1505.28	907.73
1.1	33	1401.37	1409.13	1377.47	1387.54	889.09
1.2	36	1333.73	1353.3	1318.89	1348.33	944.86
1.3	39	1269.56	1301.06	1264.3	1310.47	853.25
1.4	42	1210.99	1253.93	1215.46	1274.74	836.89
1.5	45	**1175.51**	1224.68	1183.81	1246.79	823.4
1.6	48	1178.64	**1223.95**	**1179.99**	1231.25	814.04
1.7	51	1196.22	1234	1188.97	1221.3	806.58
1.8	54	1222.91	1251.58	1207.11	1216.63	801.05
1.9	57	1253.91	1273.46	1230.62	**1216.55**	797.35
2	60	1285.89	1297.14	1256.46	1220.16	795.28
2.1	63	1296.7	1306.15	1264.58	1219.81	791.56
2.2	66	1310.01	1317.26	1275.26	1221.32	788.53
2.3	69	1325.35	1330.1	1288.1	1224.64	786.26
2.4	72	1342.2	1344.26	1302.62	1229.64	784.78
2.5	75	1360.09	1359.38	1318.38	1236.18	**784.1**
2.6	78	1378.58	1375.09	1334.95	1244.05	784.2
2.7	81	1397.31	1391.09	1351.96	1253.05	785.06
2.8	84	1415.97	1407.11	1369.09	1262.95	786.61
2.9	87	1439.97	1443.77	1386.07	1273.53	788.78
3	90	1456.15	1438.4	1402.71	1284.59	791.5

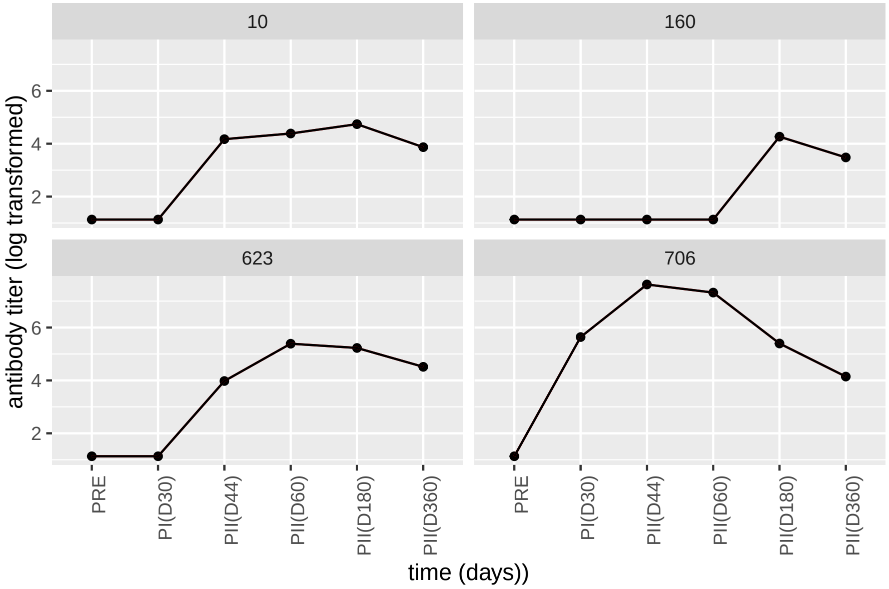

Figure S1: **Example individual fits from the Alum group with the lowest conditional AIC categorical models.** Each panel represents an individual from the Alum adjuvant group that was selected for demonstrative purposes. Red lines indicate the model fit and the black points (interconnected with black lines) indicate the measured data points. All individuals show perfect fit.

Table S2: **Marginal AIC values for 3-segment first order polynomial -restricted maximum likelihood- models on AS04 group with knot optimization.** Different first knot locations are shown in the rows and different second knot locations are shown in the columns both in months and days. NA's correspond to models that have not been built due to inconsistency between the order of knot locations. The models we have chosen are in bold fonts. All models were built by using restricted maximum likelihood.

First knot (rows) / Second knot (columns) in months		1	1.4	1.5	1.6	2	3	4
	First knot (rows) / Second knot (columns) in days	30	42	45	48	60	90	120
0.3	9	1438.84	875.3	878.55	973.35	1186.11	1278.52	1348.21
0.5	15	1439.86	876.32	879.58	974.38	1187.13	1279.54	1349.23
0.7	21	1440.53	876.99	880.25	975.05	1187.81	1280.21	1349.91
0.9	27	1441.04	877.49	880.75	975.55	1188.31	1280.72	1350.41
1.1	33	NA	877.13	887	1007.21	1203.64	1276.69	1329.94
1.3	39	NA	**874.94**	940.27	1106.69	1225.52	1255.38	1280.86
1.5	45	NA	NA	NA	1223.41	1226.63	1226.58	1235.34
1.7	51	NA	NA	NA	NA	1225.61	1219.73	1222.99
1.9	57	NA	NA	NA	NA	1223.41	1221.66	1223.57
2.1	63	NA	NA	NA	NA	NA	1227.02	1228.52
2.3	69	NA	NA	NA	NA	NA	1226.52	1228.29
2.5	75	NA	NA	NA	NA	NA	1225.85	1228.04

Table S3: **Marginal AIC values for 3-segment first order polynomial -maximum likelihood- models on AS04 group with knot optimization.** Different first knot locations are shown in the rows and different second knot locations are shown in the columns both in months and days. NA's correspond to models that have not been built due to inconsistency between the order of knot locations. The models that result in the best AIC are in bold fonts. All models were built by maximizing likelihood.

First knot (rows) / Second knot (columns) in months		1	1.4	1.5	1.6	2	3	4
	First knot (rows) / Second knot (columns) in days	30	42	45	48	60	90	120
1432.94	**865.89**	868.76	963.78	1176.97	1269.72	1339.45		
1432.94	**865.89**	868.76	963.78	1176.97	1269.72	1339.45		
1432.94	**865.89**	868.76	963.78	1176.97	1269.72	1339.45		
1432.94	**865.89**	868.76	963.78	1176.97	1269.72	1339.45		
NA	**865.89**	875.3	995.75	1192.36	1265.47	1318.5		
NA	**865.89**	930.25	1096.73	1214.68	1243.89	1268.75		
NA	NA	NA	1216.01	1216.01	1214.75	1222.6		
NA	NA	NA	NA	1216.01	1208.04	1210.14		
NA	NA	NA	NA	1216.01	1210.11	1210.69		
NA	NA	NA	NA	NA	1215.75	1215.75		
NA	NA	NA	NA	NA	1215.75	1215.75		
NA	NA	NA	NA	NA	1215.75	1215.75		

Table S4: **Marginal AIC values for 3-segment first order polynomial -maximum likelihood- models on Alum group with knot optimization.** Different first knot locations are shown in the rows and different second knot locations are shown in the columns both in months and days. NA's correspond to models that have not been built due to inconsistency between the order of knot locations. The models we have chosen are in bold fonts. All models were built by using restricted maximum likelihood.

First knot (rows) / Second knot (columns) in months		1	1.4	1.5	1.6	2	3	4
	First knot (rows) / Second knot (columns) in days	30	42	45	48	60	90	120
0.3	9	919.93	728.53	679.06	687.16	765.62	800.45	832.18
0.5	15	920.96	718.07	706.51	688.18	766.64	789.39	833.21
0.7	21	921.63	702.73	692.87	696.15	767.32	790.07	833.88
0.9	27	922.13	686.25	681.26	689.35	767.82	790.57	834.38
1.1	33	NA	685.88	**680.4**	695.49	778.86	792.88	827.29
1.3	39	NA	683.7	681.48	731.60	798.97	790.94	807.78
1.5	45	NA	NA	NA	804.80	808.01	782.67	787.75
1.7	51	NA	NA	NA	NA	806.99	778.14	781.01
1.9	57	NA	NA	NA	NA	804.79	776.55	778.50
2.1	63	NA	NA	NA	NA	NA	777.43	778.92
2.3	69	NA	NA	NA	NA	NA	776.93	778.70
2.5	75	NA	NA	NA	NA	NA	776.25	778.45

Table S5: **Marginal AIC values for 2-segment second order polynomial models.** For all these models time axis was transformed, whereas time axis values before transformation is used in the table. The models we have chosen are in bold fonts.

Knot in months	Knot in days	AS01B	AS01E	AS03A	AS04	Alum
0.5	15	1394.58	1390.78	1340.57	1295.24	792.56
1	30	1394.58	1390.78	1340.57	1295.24	792.56
1.1	33	1307.82	1324.59	1274.26	1275.54	792.65
1.2	36	1191.77	1234.98	1187.17	1228.66	776.97
1.3	39	**1189.33**	**1227.23**	**1181.72**	**1210.15**	768.1
1.4	42	1265.17	1279.7	1240.21	1213.38	**765.78**
1.5	45	1278.88	1290.04	1251.52	1215.75	766.12
2	60	1278.88	1290.04	1251.52	1215.75	766.12
3	90	1714.55	1663.81	1644.65	1513.14	912.34

Table S6: **Marginal AIC values for the categorical models.** Random intercepts in the first column show the shared or unshared random effects for the models. The first row is for a model with a separate random intercept term for each time point except $time_0$ whereas in the model shown in the last row the random intercept is unique for all time points except $time_0$. The models we have chosen and the models with the minimum marginal AIC are in bold fonts.

Random intercepts	AS01B	AS01E	AS03A	AS04	Alum
$time_1, time_2, time_3, time_4, time_5$	**495.24**	**647.01**	**653.93**	**-755.71**	**-600.75**
$time_{12}, time_3, time_4, time_5$	937.56	963.89	949.75	998.05	711.81
$time_1, time_{23}, time_4, time_5$	570.24	639.46	662.97	708.94	546.74
$time_1, time_2, time_{34}, time_5$	745.43	822.57	774.29	848.25	639.31
$time_1, time_2, time_3, time_{45}$	543.58	689.75	661.5	718.2	522.39
$time_{123}, time_4, time_5$	947.23	969.01	952.02	1004.8	731.04
$time_{12}, time_{34}, time_5$	941.62	972.2	954.51	1012.23	730.63
$time_{12}, time_3, time_{45}$	930.48	958.59	943.6	993.28	704.34
$time_1, time_{234}, time_5$	792.53	849.26	795.68	908.31	654.86
$time_1, time_{23}, time_{45}$	**578.6**	**687.06**	**669.27**	**736.6**	**542.1**
$time_1, time_2, time_{345}$	775.73	864.44	784.64	886.73	647.62
$time_{1234}, time_5$	967.65	1003.58	979.01	1013.92	739.22
$time_{123}, time_{45}$	942.08	965.65	946.24	1001.64	725.5
$time_{12}, time_{345}$	938.87	973.66	950.94	1012.46	730.51
$time_1, time_{2345}$	795.52	873.23	809.46	920.69	663.4
$time_{12345}$	965.98	1010.95	988.44	1010.46	735.43

Table S7: **Conditional AIC values for the categorical models.** Random intercepts in the first column show the shared or unshared random effects for the models. The first row is for a model with a separate random intercept term for each time point except $time_0$ whereas in the model shown in the last row the random intercept is unique for all time points except $time_0$. The models we have chosen and the models with the minimum conditional AIC are in bold fonts.

Random intercepts	AS01B	AS01E	AS03A	AS04	Alum
$time_1, time_2, time_3, time_4, time_5$	**-430.71**	**48.14**	**139.24**	**-8176.82**	**-6394.57**
$time_{12}, time_3, time_4, time_5$	793.11	808.91	802.39	827.01	590.83
$time_1, time_{23}, time_4, time_5$	13.74	55.35	199.45	213.96	302.15
$time_1, time_2, time_{34}, time_5$	392.6	500.98	445.71	502.67	493.08
$time_1, time_2, time_3, time_{45}$	-81.92	213.99	196.2	233.01	193.4
$time_{123}, time_4, time_5$	824.93	830.77	820.31	850.7	632.21
$time_{12}, time_{34}, time_5$	816.34	836.41	820.44	864.16	641.11
$time_{12}, time_3, time_{45}$	794.66	814.57	808.75	833.52	591.84
$time_1, time_{234}, time_5$	534.67	583.58	507.93	663.12	542.55
$time_1, time_{23}, time_{45}$	**47.2**	**228.26**	**231.13**	**288.58**	**312.09**
$time_1, time_2, time_{345}$	490	631.41	502.49	616.12	528.02
$time_{1234}, time_5$	879.35	900.98	862.35	885.66	667.97
$time_{123}, time_{45}$	826.39	836.67	820.96	856.73	633.14
$time_{12}, time_{345}$	823.51	851.95	824.06	879.51	653.69
$time_1, time_{2345}$	572.56	671.44	556.73	715.06	571.31
$time_{12345}$	882.39	915.59	879.08	886.4	668.24

Table S8: **BIC values for the categorical models.** Random intercepts in the first column show the shared or unshared random effects for the models. The first row is for a model with a separate random intercept term for each time point except $time_0$ whereas in the model shown in the last row the random intercept is unique for all time points except $time_0$. The models we have chosen and the models with the minimum BIC are in bold fonts.

Random intercepts	AS01B	AS01E	AS03A	AS04	Alum
$time_1, time_2, time_3, time_4, time_5$	**580.30**	**-772.34**	740.08	**-745.97**	**-475.09**
$time_{12}, time_3, time_4, time_5$	1003.28	1028.98	1014.99	1063.58	771.54
$time_1, time_{23}, time_4, time_5$	635.96	704.55	728.20	774.47	606.46
$time_1, time_2, time_{34}, time_5$	811.16	887.67	831.43	913.78	699.04
$time_1, time_2, time_3, time_{45}$	1037.75	1136.76	1139.67	1280.706	882.39
$time_{123}, time_4, time_5$	997.49	1018.78	1002.06	1054.92	776.71
$time_{12}, time_{34}, time_5$	991.88	1021.98	1004.39	1062.34	776.30
$time_{12}, time_3, time_{45}$	980.74	1008.36	991.90	1043.39	750.01
$time_1, time_{234}, time_5$	842.80	899.03	845.57	958.42	700.53
$time_1, time_{23}, time_{45}$	**628.86**	**736.84**	**719.15**	**786.72**	**587.76**
$time_1, time_2, time_{345}$	825.99	914.21	834.52	936.84	693.29
$time_{1234}, time_5$	1006.31	1041.86	1017.38	1052.46	774.34
$time_{123}, time_{45}$	980.74	1003.93	984.62	1040.18	760.62
$time_{12}, time_{345}$	977.53	1011.94	989.31	1051.00	765.64
$time_1, time_{2345}$	834.18	911.51	847.84	959.23	698.53
$time_{12345}$	996.91	1041.57	1019.14	1041.30	763.54

Table S9: **DIC values for the categorical models.** Random intercepts in the first column show the shared or unshared random effects for the models. The first row is for a model with a separate random intercept term for each time point except $time_0$ whereas in the model shown in the last row the random intercept is unique for all time points except $time_0$. The models we have chosen and the models with the minimum DIC are in bold fonts.

Random intercepts	AS01B	AS01E	AS03A	AS04	Alum
$time_1, time_2, time_3, time_4, time_5$	**451.24**	**603.01**	**609.93**	**-799.71**	**-644.75**
$time_{12}, time_3, time_4, time_5$	903.56	929.89	915.75	964.05	677.81
$time_1, time_{23}, time_4, time_5$	536.24	605.46	628.97	674.94	512.74
$time_1, time_2, time_{34}, time_5$	711.43	788.57	740.29	814.25	605.31
$time_1, time_2, time_3, time_{45}$	509.58	655.75	627.5	684.2	488.39
$time_{123}, time_4, time_5$	921.23	943.01	926.02	978.8	705.04
$time_{12}, time_{34}, time_5$	915.62	946.2	928.51	986.22	704.63
$time_{12}, time_3, time_{45}$	904.48	932.59	917.6	967.28	678.34
$time_1, time_{234}, time_5$	766.53	823.26	769.68	882.31	628.86
$time_1, time_{23}, time_{45}$	**552.6**	**661.06**	**643.27**	**710.6**	**516.1**
$time_1, time_2, time_{345}$	749.73	838.44	758.64	860.73	621.62
$time_{1234}, time_5$	947.65	983.57	959.01	993.92	719.22
$time_{123}, time_{45}$	922.08	945.65	926.24	981.64	705.5
$time_{12}, time_{345}$	918.87	953.66	930.94	992.46	710.51
$time_1, time_{2345}$	775.52	853.23	789.46	900.69	643.4
$time_{12345}$	949.98	994.95	972.44	994.46	719.43

Table S10: **Conditional corrected AIC values for the categorical models.** Random intercepts in the first column show the shared or unshared random effects for the models. The first row is for a model with a separate random intercept term for each time point except $time_0$ whereas in the model shown in the last row the random intercept is unique for all time points except $time_0$. The models we have chosen and the models with the minimum conditional corrected AIC are in bold fonts.

Random intercepts	AS01B	AS01E	AS03A	AS04	Alum
$time_1, time_2, time_3, time_4, time_5$	**-433.09**	48.76	**156.99**	**-8178.47**	**-6397.46**
$time_{12}, time_3, time_4, time_5$	816.12	825.54	830.7	851.55	613.84
$time_1, time_{23}, time_4, time_5$	27.84	**36.77**	208.56	224.41	303.32
$time_1, time_2, time_{34}, time_5$	404	510.87	442.9	510.06	505.03
$time_1, time_2, time_3, time_{45}$	-99.65	219.75	204.54	239.82	188.18
$time_{123}, time_4, time_5$	819.35	824.61	840.41	846.75	632.95
$time_{12}, time_{34}, time_5$	817.64	831.49	825.73	858.26	640.28
$time_{12}, time_3, time_{45}$	793.46	810.61	823.27	828.69	594.61
$time_1, time_{234}, time_5$	516.11	565.68	485.98	639.91	542.42
$time_1, time_{23}, time_{45}$	**43.57**	**226.66**	**228.93**	**287.49**	**303.51**
$time_1, time_2, time_{345}$	472.98	611.52	502.45	599.31	527.41
$time_{1234}, time_5$	887.86	906.58	870.28	894.38	674.44
$time_{123}, time_{45}$	807.48	815.97	823.09	836.64	620.79
$time_{12}, time_{345}$	801.89	829.74	816.51	866.9	645.48
$time_1, time_{2345}$	574.38	668.37	555.16	707.23	578.68
$time_{12345}$	878.89	913.79	878.67	886.25	663.94
